# From elemental qualities to medication: Hippocratic pharmacological system in classical Greek medicine

**DOI:** 10.3389/fphar.2026.1820951

**Published:** 2026-04-15

**Authors:** YanLiang Fan, Wenjun Wu

**Affiliations:** 1 Faculty of History, Nankai University, Tianjin, China; 2 School of Marxism, Jimei University, Xiamen, China

**Keywords:** ancient Greece, *Hippocratic Corpus*, history of medicine, pharmacology, philosophy of medicine

## Abstract

While numerous scholars have studied Hippocrates’ philosophy, ideas, and oaths, there has been limited research on his pharmacological theories especially combined with clinical medication. This paper extracts and reconstructs a pharmacological system from the extensive corpus of Hippocrates, dividing it into two parts: the first is the philosophical theory of medicine, exploring the natural properties of all things and humans, producing the Four Humors Theory; the second is clinical pharmacology, discussing how to maintain balance to preserve or restore health. The system comprises two major inferences: first, the four elemental qualities form the foundational logic of natural philosophy and the pharmacological essence of medical philosophy; second, the system guides the complementary “regimen therapy” and the “evacuation therapy” based on the underlying logic of the four elemental qualities. Through theoretical construction and practical validation, this reflects ancient Greece’s exploration of the relationship between pharmacal practice and medical philosophy during the classical era.

## 1 Introduction

The ancient Greek medicine documented in written records was initially imbued with mystique and romanticism, as evidenced by descriptions of wound treatment (Homer, 1919; Homer, 1924) and oral herbal infusions (Homer, 1919) in the *Homeric Epics*. Subsequently, medical practices in ancient Greece were predominantly temple-centered, with therapies often relying on dream therapy and empirical formulas. After the seventh century BCE, the rise of natural philosophy in ancient Greece saw philosophical doctrines proposed by Anaximander, Pythagoras, Empedocles and etc., exerting certain influences on numerous medical practitioners, including Hippocrates.

Anaximander was the first to employ two pairs of opposing natural qualities—cold (ψυχρός) and hot (θερμός), dry (ξηρός) and moist (ὑγρός)—to explain how the unlimited (ἄπειρον) gives rise to all things, how they change, and ultimately returns to the unlimited (Burnet, 1920; Laks and Most, 2016). The use of these four natural qualities (hereafter referred to as the “four elemental qualities”) to explain the changes and interactions of things may have provided inspiration for Hippocrates’ pathology and pharmacology. Pythagoras believed that all things were governed by numbers (Laks and Most, 2016), and the tertians, quartans, quintans, septans, nonans, as well as “crisis” (κρίσις)^1^ described in *Epidemics I* were likely influenced by the Pythagorean School (Hippocrates, 1923a). Pythagoras was also the first to emphasize the harmonization of numbers. Then Alcmaeon drew upon Pythagorean idea of harmony and proposed a philosophical explanation closest to that of Hippocrates: the state of health is achieved when the qualities within the body—such as cold and hot, dry and moist, bitter and sweet—are all in balance; An imbalance in any of these aspects would cause physical weakness and even illness (Laks and Most, 2016). Empedocles proposed the “theory of four elements,” asserting the origin of all things was fire (πῦρ), water (ὕδωρ), earth (γῆ), and air (ἀήρ) (Laks and Most, 2016). This theory, along with its predecessor, also exerted a certain influence on Hippocrates’ medical philosophy.

Hippocrates was the most renowned physician in ancient Greece. Plato regarded Hippocrates as the epitome of physicians of his era, just as Phidias of Athens and Polyclitus of Argos were celebrated as master sculptors (Plato, 1924). Aristotle once observed: “Hippocrates’ greatness lies not in his stature, but in his exceptional medical genius” (Aristotle, 1932). Currently, over sixty medical works written in the Ionian dialect are attributed to Hippocrates, and scholars collectively refer to these works as the *Hippocratic Corpus* (hereinafter referred to as the *Corpus*). The *Corpus* is evidently not the work of a single author, just as the “The Homer Question” exists, so too does the “The Hippocratic Question” (Lloyd, 1975; Smith, 1979). Although some texts are believed by scholars to have been authored by Hippocrates’ students Polybius^2^ and Syennesis, the discussion on “The Hippocratic Question” ultimately reached a stalemate (Jouanna, 2023). For convenience, this article will set aside “The Hippocratic Question” and use the term “Hippocrates” in a broad sense, which can refer to both a historical figure and serve as a collective designation for the authors of the various chapters in the *Corpus*.

The *Corpus* not only contains professional medical knowledge and skills, but also natural philosophy, medical thought, medical ethics, and doctor-patient relationships, which are highly valued by modern researchers (Jouanna, 1999; 2012; Longrigg, 1998; Phillips, 1973). However, most current researches study medical philosophy and medical technics (τέχνη) separately, each in isolation, failing to establish a profound connection between theory and practice. So there remains room for supplementation and revision in the discussion and construction of a more comprehensive pharmacological system.

However, elucidating this connection presents a structural challenge for this article. So there will be lacks on clinical evidence in the theoretical statement, but the callbacks will be presented in the subsequent practical chapters. In this way, a pharmacological system with a coherent beginning and end could be established, just like the structure of Tai Chi diagram. This method also aligns with the dialectical relationship between theory and practice as articulated in the Hippocratic tradition: The theory guides the practice, but the latter also generates feedbacks on the former. In addition, a pharmacal gradient therapy framework with deepening levels will be built on the basis of simple classification by Hippocrates. Furthermore, a limited comparison method will be used between Hippocratic medication and Traditional Chinese Medicine (TCM) to further elucidate and prove the clinical efficacy of drugs.

Given the current research status, the focus of this paper does not concentrate on the philosophy or technic itself but a pharmacological system simultaneously containing medical philosophy, pharmacal gradient therapy framework and clinical medication, so as to reflect the ancient Greeks’ exploration of the relationship between pharmacal practice and medical philosophy during the classical era.

## 2 Hippocrates’ medical philosophy and preliminary construction of gradient therapy

The discussions on medicinal substances in the *Corpus* are highly fragmented, with their applications closely tied to specific medical topics. However, the entire *Corpus* shares a common underlying logic in the use of internal medicine, namely, the four elemental qualities. This fundamental logic can be understood from two perspectives within Hippocratic medical philosophy: First, as one of the sources of four humors, the four elemental qualities possess natural universality, commonality, and fluidity, serving as the foundation of Hippocratic natural philosophy and the underlying logic of the humorism (physiology). Second, physicians can utilize the commonality and fluidity of the four elemental qualities to counteract external substances that disrupt the balance of the four humors, which constitutes the essence of Hippocratic pharmacology. The application of this logic in clinical practice is categorized by Hippocrates into two approaches: replenishment (regimen) and evacuation, thereby giving rise to two major categories of Hippocratic internal medicine—regimen drugs and evacuative drugs. This classification forms the basis of Hippocratic gradient therapy.

### 2.1 Four elemental qualities: the basic logic of Hippocratic pharmacology

The Four Elemental Qualities, which is closely tied to ancient Greek natural philosophy, is the basic logic and one of the origins of the Four Humors Theory. Many philosophers and physicians of Hippocrates’ era attempted to explain the composition of human body using elemental theories, which exerted a profound influence in ancient Greece. But Hippocrates argued that water, fire, earth, and air were clearly not components of the human body (Hippocrates, 1931). Some physicians used medical concepts to explain the elemental component of body, with some attributing it to blood, others to bile, and still others to phlegm. The common thread among these theories was the belief that the human body was composed of a single elemental component^3^. The term “elemental component” was expressed differently in various passages of the *Corpus*, such as *ἐνεὸν*, *ἕν*, *ἐόντα*, *ἑνός* and *ἐνεόντα*. The prototypes of *ἕν* and *ἑνός* are *εἷς* (one), while *ἐόν* and *ἐόντα* are *εἰμί* (being), making *ἐνεόν* (*ἕν ἐόν*) and *ἐνεόντα* (*ἕν ἐόντα*) literally mean indivisible existence. These terms in the original text all refer to a single, indivisible, and universal philosophical entity (πᾶν). And the concept was not first proposed by Hippocrates; many philosophers, when describing their own origins in natural philosophy, favored similar terms and variations (Laks and Most, 2016). According to Hippocrates, a single elemental component alone cannot cause disease, otherwise the treatment should be singular, yet in practice multiple therapies do exist. Hippocrates explained the diversity of etiologies and treatments as follows: for the human body contains various components, diseases are triggered by the influence of opposing natural properties such as the hot, the cold, the dry, or the moist; so that both the forms of diseases are numerous and the corresponding treatments are manifold (Hippocrates, 1931).

According to *Nature of Man* (*ΠΕΡΙ ΦΥΣΙΟΣ ΑΝΘΡΩΠΟΥ*), a single component cannot generate all things; only when they are mutually coordinated and no fewer than two are present is it possible. This is both the nature of all things and the human beings. A human cannot be composed of a single component, but rather contain various constituents with multiple elemental qualities that collectively contribute to body. Again, each component must return to its own nature when the body of man dies, moist to moist, dry to dry, hot to hot, and cold to cold. Such too is the nature of animals, and of all other things. Thus, all things are born in a like way and die in a like way, with the starting point also being the final destination (Hippocrates, 1931). Thus, the four elemental qualities transcend the scope of the human body itself, possessing natural universality, commonality, and fluidity. In the view of natural philosophy, even without introducing the concept of four humors, both pathology and pharmacology can be explained through the four elemental qualities. Therefore, the four elemental qualities can be considered the underlying logic of Hippocratic natural philosophy.

However, as a physician, given the universality of the four elemental qualities in nature, to highlight the uniqueness of humans and the refined theoretical demands of clinical practice, and in conjunction with the contemporary medical theories of blood primacy, phlegm primacy, and bile primacy, Hippocrates attempted to construct a “Four Humors Theory” based on Empedocles’ concept of the natural “Four Elements.” Hippocrates posited that the human body consists of blood, phlegm, yellow bile, and black bile (Hippocrates, 1931). These components are continuously consumed and produced within the body, maintaining a certain state of balance (Hippocrates, 1931). The Four Humors Theory formed the foundation of Hippocrates’ physiology, closely linking human health and disease: the body enjoys the most perfect health when these elements are duly proportioned to one another in respect of compounding, power and bulk, and when they are perfectly mingled; disease and pain occur when one of these elements is in defect or excess, or is isolated in the body without being compounded with all the others (Hippocrates, 1931).

It is evident that Hippocrates “packaged” four elemental qualities as four humors, thereby transforming their properties into material substances, and also aligning more closely with physicians’ hypotheses regarding human composition. Furthermore, Hippocrates’ division of bile into the yellow and the black reflects his borrowing of Empedocles’ integration of four elements, making the result that four humors are more consistent with the numerical logic of the four elemental qualities too. These philosophical and medical concepts, notions of harmony and balance, and the idea of numbers all demonstrate Hippocrates’ inheritance and continuation of earlier philosophical and medical achievements. Additionally, based on Hippocrates’ descriptions of the relationship between humoral balance and health status, it is clear that the four humors replaced the pathogenesis mechanism of the four elemental qualities, acting as carriers that encapsulate the four elemental qualities within the humors.

**FIGURE 1:**
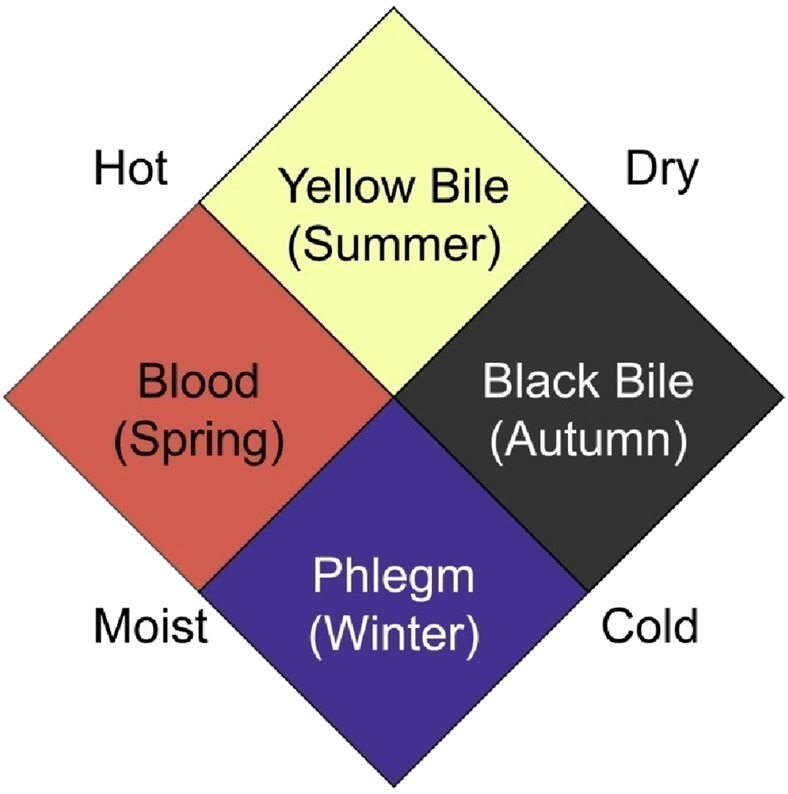
The correspondence between the four humors and the four elemental qualities.

The correspondence between the four humors and the four elemental qualities (Figure 1) can be deduced from the descriptions of seasonal variations in humoral balance in the *Corpus*:

Spring being moist and warm, the quality of this season in the year is most akin to the nature of blood … In summer, phlegm is at its weakest, as summer is opposed to its nature, being dry and hot … Bile prevails in the body during the summer season and autumn … In summer, human vomitus is most yellow-bilious … For autumn, it is dry and induces a feeling of coolness, black bile is greatest and strongest at this time and blood becomes least … In autumn, human vomitus is most black-bilious … Phlegm, being the coldest constituent of the body, is closest akin to winter … In winter, human vomitus is most phlegmatic (Hippocrates, 1931).

It is obvious that one humor regularly corresponds to two elemental qualities, and considering both the theory of elemental qualities and the humoral theory in health and disease, we can conclude the four elemental qualities are the underlying logic of the four humors. Additionally, it can be observed that the four humors fluctuate with the seasons, waxing and waning in a cyclical manner. This is due to climatic changes causing the four humors/elemental qualities in the body to move equilibrium to adapt to seasonal variations, reflecting the fluidity of the four elemental qualities too.

It should be noted that unlike Galen, Hippocrates did not fully correlate the four elements with four humors and four elemental qualities. Only incomplete clues could be found in *On Regimen* (*ΠΕΡΙ ΔΙΑΙΤΗΣ*):

All animals, including man, are composed of two things, different in power but working together in their use, namely, fire and water … Fire can move all things always, while water can nourish all things always … Fire has the hot and the dry, water the cold and the moist (Hippocrates, 1931).

The author of *On Regimen* believed that the human body is composed of fire and water, while the author of *Nature of Man* ^4^ proposed the Four Humors Theory and explicitly opposed the elemental theory, which is an evidence that different chapters in the *Corpus* were authored by different individuals and were created at distinct times. However, the dualism of water and fire also explains changes through the flow and interaction of four elemental qualities (Hippocrates, 1931). Thus, whether it is the four humors or the two elements that constitute human body, their interactions with each other inside and with substances outside are all based on the four elemental qualities, without affecting Hippocrates’ philosophical system or pharmacal practice. As for the other two elements, air and earth, according to the surviving *Corpus*, they were not associated with human body by Hippocrates. Although Galen in the second century AD was convinced that the connection between the four elements and the four humors originated from Hippocrates, claiming him as the first to propose that the human body is composed of water, fire, earth, and air. However, the academic community nowadays widely holds that the theory of the four elements in human physiology originated with Empedocles. Galen further explained that the four elements function through the four elemental qualities, too (Galen, 1999). This concept partially derives from Hippocrates’ dualism of water and fire in his *On Regimen*, while the other may have been inspired by Aristotle’s correspondence between the four elements and the four elemental qualities. These show the harmonizing role of the four elemental qualities in resolving contradictions and their influence on later medicine, and both benefited from and proved the four elemental qualities to be the foundation and underlying logic of Hippocratic philosophy. Thus, when explaining the interactions between external factors and human body, especially the therapeutic effects of drugs on patients, the four elemental qualities re-emerges from four humors, reaffirming its original significance.

### 2.2 The rudiment of gradient therapy: regimen and *pharmacon* based on four elemental qualities

Hippocrates posited that most natural or artificial substances possess certain pharmacological therapeutic effects (Hippocrates, 1923b). This is attributed to the universality of four elemental qualities, where different substances inherently exhibit distinct properties of cold or hot, dry or moist, with each property further differentiated (e.g., hot and warm). This provides a rich material basis for pharmacological interventions, enabling the treatment of diverse pathological conditions. According to the humoral theory, disease arises from the disruption of original humoral balance, as well as the balance of four elemental qualities. To restore it, the *Breaths* (*ΠΕΡΙ ΦΥΣΩΝ*) states: “Medicine in fact is subtraction and addition, subtraction of what is in excess, addition of what is wanting. He who performs these acts best is the best physician; he who is farthest removed therefrom is also farthest removed from the art” (Hippocrates, 1923b). Theoretically, if a patient suffers from excessive dryness and heat with insufficient moisture and cold, medications with moist and cold properties should be administered to counteract the opposites, thereby restoring balance, which is Hippocrates’ addition of deficiency. But if the dryness and heat are too severe to counteract through dosage adjustments, evacuative therapies are considered to eliminate the excess dryness and heat, which is Hippocrates’ subtraction of excess. Thus, the four elemental qualities serve as a bridge connecting drugs and the human body (commonality), functioning as a medium for material exchange and interaction between the body and the external environment (fluidity) whether through “addition” or “subtraction.” This constitutes the essence of Hippocratic pharmacology.

As a result, in *Nature of Man*, Hippocrates classified therapeutic methods into two categories— “regimen (δίαιτα)”^5^ and “pharmacal therapy (φάρμακον)” separately based on the principles of addition and subtraction: “This one should learn and change, and carry out treatment only after examination of the patient’s constitution, age, physique, the season of the year and the fashion of the disease, sometimes taking away and sometimes adding, as I have already said, and so making changes in pharmacon or in regimen to suit the several conditions of age, season, physique and disease” (Hippocrates, 1931). Phillips thus concluded that Hippocrates’ therapeutics consisted of two types: one being a mild dietary and exercise regimen for the health, termed “Regimen;” and the other involving more active intervention during the course of disease, termed “Therapy” (Phillips, 1973). But this may be not accurate.

Regimen was the most highly recommended treatment method by Hippocrates (Hippocrates, 1923b), not only for diet and exercise but therapy, and its essence lies in a single sentence, “opposites are cures for opposites” (Hippocrates, 1923b). *The Sacred Disease* (ΠΕΡΙ ΙΕΡΗΣ ΝΟΥΣΟΥ) states: “Whoever knows how to cause in men by regimen dry or moist, cold or hot, he can cure this disease also, if he can distinguish the seasons for useful treatment, without resorting to purifications and magic” (Hippocrates, 1923b). This demonstrates Hippocrates’ emphasis on regimen and the essential role of the four elemental qualities in this approach. It is evident that regimen can treat diseases by regulating the body’s elemental or humoral states, extending beyond mere health maintenance for healthy individuals, thus encompassing both dietary (food) regimen and pharmacal (drug) regimen. Hippocrates’ advocacy for regimen is further reflected in his criticism of physicians who over-relied on evacuants while neglecting regimen (Hippocrates, 1923b). This orientation preliminarily reveals Hippocrates’ gradient therapy: for the health or sub-health, he favored dietary regimen; for patients with mild conditions, he employed mild therapeutic pharmacal regimen; and for severe cases unresponsive to these methods, he resorted to more aggressive therapies like emesis or purgation. Compared to the regimens in two different conditions, evacuative therapy clearly showed lower patient compliance^6^. However, compared to the limited evacuative medications, the therapeutic of regimen required mastering a vast array of natural agents and achieving more precise disease diagnosis and treatment, which also imposed higher technical demands on the physicians at that time.

In the *Corpus*, the term *pharmakon* carries Hippocratic connotations, specifically referring to substances with evacuative effects such as emetics and purges. As previously mentioned, regimen can also treat diseases, indicating that the substances used in regimen align with the concept of “drugs” in modern time. In other words, Hippocratic medication encompasses two clinical scenarios: one is the relatively mild “drugs for regimen”, and the other is the more potent “drugs for evacuation” used to purge, vomit, sweat, or sneeze. If used to preserve health in healthy individuals, it is still referred to as “food”.

## 3 Hippocrates’ practice of regimen

In Hippocrates’ clinical medical practice, the regimen best exemplifies the philosophical logic of the Four Elemental Qualities. This article will provide a detailed explanation using seasonal healthy diet and three clinical treatment cases as examples of food regimen and drug regimen.

### 3.1 Dietary regimen in seasons for healthy individuals

\In ancient Greece, summers were scorching and arid, while winters were bitterly cold and damp. The body’s humoral balance shifted with seasonal changes. To counteract these variations, Hippocrates proposed a health-preserving principle: dietary patterns should align with the seasons to maintain wellness and prevent diseases. The relationship between regimen practices and philosophical theories follows a logical chain: practical observation → philosophical characterization → connection to the Four Elemental Qualities Theory → practical application for health guidance → observation of outcomes → verification or revision of theory based on results.

Winter is characterized as cold and moist, during which phlegm predominates in the balance of the four humors. To prevent excessive phlegm from disrupting humor equilibrium and causing illness, Hippocrates advocated for foods and beverages with dry and warm properties. For instance, vegetables should be dry and warm, and roasted meat is preferable to boiled meat. It is hypothesized that different cooking methods can alter the properties of food to varying degrees. For instance, grilling may reduce the moisture in food, while soaking or boiling can mitigate its inherent dryness. Dark wine or slightly diluted wine is considered optimal. It is hypothesized that the more concentrated and darker the color of (red) wine, the drier and hotter its properties usually tend to be. The ancient Greeks had the custom of diluting wine with water; the more diluted and lighter the color, the more it could mitigate the dry and hot characteristics, which Hippocrates described as becoming “milder”. Due to the moist of winter and its alignment with the nourishing properties of water, it is prone to have excessive food retention. Therefore, emetics are to be used three times a month by those with a moist constitution, while twice a month by dry constitutions (Hippocrates, 1931).

Spring is characterized by warm and humid weather, classified as moist-hot in Hippocratic medicine. The approaching summer, however, brings dry and hot. Thus, the dietary regimen of spring focuses on transitioning from winter to summer. The diet in spring should appropriately incorporate moist-inducing foods, such as boiled vegetables, and maintain a balanced combination of boiled and roasted meats. Wine gradually becomes lighter in color and thinner in concentration. Spring regimen should be gentle, nourishing the body until summer arrives (Hippocrates, 1931).

During the hot and dry summer season, the body’s phlegmatic content reaches its lowest level. Seasonal dietary intake should prioritize mild, moist-cold properties, avoiding dry-hot foods and undiluted dark beverages. Boiled vegetables is recommended to mitigate dryness, while wine should be light in color and flavor. This season aligns with the fire element’s characteristic of consumption, emphasizing energy expenditure. Excessive bodily depletion should be avoided, and unless dietary intake is excessive, emetics are generally not advisable (Hippocrates, 1931).

Autumn is characterized by dryness and a gradual decline in temperature, classified as dry-cold in nature. Similar to spring, autumn dietary regimen adopts a gradual transition from summer to winter in all aspects to avoid abrupt dietary changes. Foods should be warm and low in moisture; drinks should gradually darken in color and preferably not be diluted with water. Vegetables should primarily be dry in nature (Hippocrates, 1931).

The analysis of Hippocrates’ seasonal regimen yields the following conclusions: Firstly, the theoretical guidance of it originates from the philosophical logic of four elemental qualities. Secondly, seasonal regimen embodies Hippocrates’ concept of using food as “medicine,” sharing similar objectives with “ZhiWeiBing” (means to cure the disease in advance in TCM, namely, preventive treatment) and Modern Preventive Medicine. Additionally, it is evident that the Four Elemental Qualities in clinical practice are not limited in the antagonistic logic of oppositional therapy.

The principle of complementary opposition is particularly evident in the regimen of summer and winter, while the spring and autumn regimen do not simply oppose seasonal characteristics. Instead, they adopt a gentler approach that partially adapts to seasonal traits and evolutionary trends. For instance, during the moist-hot spring, regimen aligns with the season’s moist nature, transitioning from the dry winter regimen to a moist diet, gradually evolving toward the summer’s moist regimen. Autumn regimen follows a similar pattern, the dry or less moist diets accommodate the season’s dryness. Taking the dry-cold autumn as an example, if a completely opposite regimen were applied—as a shift from the summer’s moist-cold regimen to the winter’s dry-hot regimen—the moist-hot regimen can serve as a transitional approach theoretically. However, Hippocrates adopted a partially adaptive dry-warm regimen for autumn, likely influenced by clinical observations, thereby modifying theory to align with practice. This might be interpreted as a theoretical explanation: as summer wanes, a dry-warm regimen that aligns with summer’s characteristics, which would also apply to winter, albeit more mild (the warm differing from the hot).

The practice of seasonal dietary regimen reflects Hippocrates’ non-conformist thinking that transcends theoretical rigidity, while upholding the pragmatic approach of medical practice. The gradual evolution of spring or autumn regimen between summer and winter demonstrates that external interventions should avoid drastic changes during the seasonal shifts in humoral balance. Nevertheless, the Four Elemental Qualities Theory continues to serve as the foundational logic for theoretical interpretation.

### 3.2 Pharmacal regimen for patients

Beyond guiding the diet for health maintenance of healthy individuals, Regimen can also serve as a clinical medication therapy for patients. When describing the properties of a medicinal substance, Hippocrates typically employed the model of “four elemental qualities + effects on evacuation” (Hippocrates, 1931). This indicates that in the application of pharmacal regimen, beyond the interactions of the four elemental qualities, Hippocrates also considered the potential, more mild evacuative effects of a medicinal substance compared to the evacuants.

In addition to therapeutic ingredients, the *Corpus* primarily documented four oral dosage forms in pharmaceutics: cyceon (κυκεών)^7^, wine, hydromel, and herbal water. Among these, compound preparations from wine and hydromel (excluding barley) usually contained no more than three components^8^, whereas cyceon and herbal water supported more complex ingredients.

#### 3.2.1 Case 1: child urinary calculi

Case in Text: If a child develops urinary calculi due to consuming unhealthy, excessively hot and bilious milk, Hippocrates proposed feeding the child only with wine heavily diluted with water, as this would minimize the heat effect on vessels (Hippocrates, 1923a).

Analysis: The two primary elemental qualities causing excessive urinary calculi in children are the hot and dry (bilious) of milk (Hippocrates, 1931). In Hippocrates’ prescriptions, the water is moist and cold, the pure wine dry and hot (Hippocrates, 1931), diluting the wine significantly by water, thereby reduced the dry-hot effect and minimized its impact on vessels. Using dry-hot wine as the base is intended to prevent overly polarized therapeutic approaches (refer to the spring and autumn regimen), which may hinder harmonization. The integration of water’s moist-cold and wine’s dry-hot facilitates more effective elemental flow and harmonization with the excess dry-hot in the child’s body.

#### 3.2.2 Case 2: patient with ocular pains

Case in Text: In treating ocular pains, Hippocrates suggested drinking neat wine, bathing in copious hot water, and bleeding (Hippocrates, 1931).

Analysis: In this case, Hippocrates did not directly give an explanation with the theory of four elemental qualities. Based on descriptions of eyelid heaviness, body pain, and headaches in *On Regimen* (Hippocrates, 1931), combined with therapies like “neat wine, hot baths, and bloodletting,” it is inferred that the pains in patient’s eyes resulted from food excess and lack of exercise leading to food accumulation, which in turn caused excessive moisture—likely moist-hot or moist-cold, with the moist being the primary cause and hot or cold the secondary factors. Therefore, drinking pure wine was due to its dry, and bloodletting was to reduce the moist. If the cause was moist-hot, this would be even more applicable. Bathing exposes the body to external moisture, though less impactful than internal consumption. Thus, physician advised the patient to take hot baths to alleviate the moist.

#### 3.2.3 Case 3: patient with malaria

Case in Text: The person suffered the following: the patient’s complexion was pale-yellow below the eyes. He expectorated pale-yellow sputum, and when an attack occurred he choked and sometimes cannot cough even though he wanted to. Sometimes, because of his choking and eagerness to cough, he all at once vomited bile, then scum, and often even food when he had eaten. Shortly after vomiting, the patient felt somewhat better, but soon relapsed into the same distress as before. His voice became shriller than when he was well, and intermittent chills and fever accompanied by sweating occurred (Hippocrates, 1988).

Hippocrates’ Comments: His spinal marrow became filled with blood. He may also be consumed, as the hollow vessels fill with dropsical phlegm and with bile. Both of these are sources of consumption, causing the same symptoms in the patient (Hippocrates, 1988).

Hippocrates’ Prescription: In the third month, Hippocrates prescribed a cyceon to the patient, flavoured with celery roots (σέλινον, *Apium graveolens* L.), dill (ἄνηθον, *Anethum graveolens* L.), rue (πήγανον, *Ruta graveolens* L.), mint (μίνθη, *Mentha viridis* (L.) L.), coriander (κορίαννον, *Coriandrum sativum* L.), fresh poppies (μήκων, *Papaver somniferum* L.), basil (ὤκιμον, *Ocimum basilicum* L.), lentil (φακός, *Ervum lens* L.), and the juices of sweet and vinous pomegranates (ῥοιή, *Punica granatum* L.), the amount of the sweet being double that of the vinous. The entire preparation was as follows. Prepare a mixture of half a cotyle (κοτύλη)^9^ of two pomegranate juices together, half a cotyle of pleasant dry dark wine, and half a cotyle of water. Grind the aforementioned plants fine, then soak them in the mixture, and pour into a cup. Next, add vetch-meal (ὄροβος, *Vicia ervilia* (L.) Willd.) with the amount of 1 oxybaphon (ὀξύβαφον)^10^, an equal amount of barley meal, and grate in an equal amount of aged goat’s cheese (τυρός). Stir well. After the cyceon is cooked, have the patient drink it off (Hippocrates, 1988).

Analysis: Based on the patient’s symptom description, it is difficult for modern practitioners to provide a etiological explanation with four elemental qualities theory. However, by analyzing key terms such as “intermittent chills and fever accompanied by sweating,” it is highly probable that the condition is attributed to plasmodium infection, i.e., malaria. The typical symptoms of malaria, in addition to intermittent fever, chills, shivering, and sweating, often include physical signs such as fatigue, headache, and limb pain. Accompanying symptoms may also manifest as cough, anorexia, vomiting, diarrhea, weakness, sore throat, and hoarseness, which generally align with Hippocrates’ description of the patient’s symptoms. In response, Hippocrates attributed the etiology to excessive blood consumption, leading to an overabundance of phlegm and bile. The excessive deposition of these substances in the blood vessels accelerates the aging of newly formed blood, ultimately transforming it into phlegm, thereby sustaining or even exacerbating the predominance of phlegm and bile. Prior to the third month of regimen therapy, the original text mentions that if the patient in the first month refuses to adhere to complex dietary, exercise, bathing, or even bedding material regimens, emesis therapy may be considered (Hippocrates, 1988). This demonstrates the diversity and flexibility of Hippocrates’ therapeutic approach, as well as his medical ethics in providing patients with options. Additionally, the progression from severe to mild conditions, from the first month’s emesis therapy to the third month’s regimen therapy, reflects a gradient in pharmacal treatment.

**FIGURE 2:**
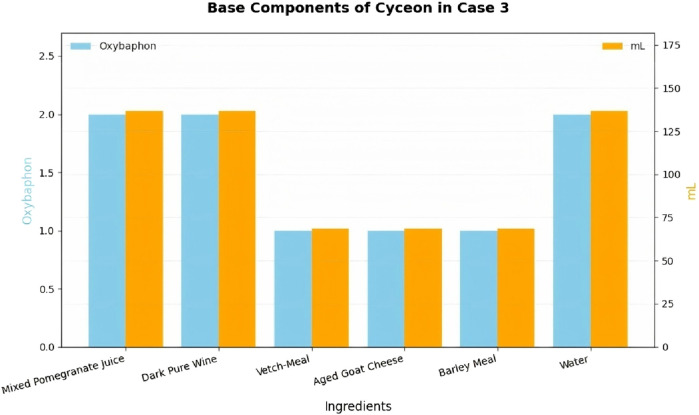
The pharmaceutical base components of cyceon used by Hippocrates in Case 3.

In the third month of the treatment course, Hippocrates prescribed a cyceon with a mixed pharmaceutical base (Figure 2).

The cyceon, when combined with wine, exhibits a warm and nutritious nature, while the addition of goat cheese has a mild laxative effect; The boiled-down wine warms, moistens and sens to stool; The goat cheese is heating and nourishing; Sweet pomegranate juice is laxative, having a certain burning quality; And vetch provides nourishing and strengthening properties (Hippocrates, 1931). This demonstrates that the base of the compound decoction is predominantly characterized by moist-hot, aiming to supplement and enhance the patient’s blood nature. The mild laxative effect facilitates smooth gastrointestinal function, aiding in bowel movements and clearing accumulated phlegmatic and bilious components.

Regarding the herbal components (Table 1), They are primarily warm in nature, which helps strengthen the blood; some are laxative to ensure that the excretion pathways of phlegmatic and bilious components are not blocked; the ingredients for relieving coughs, suppressing coughs, and preventing vomiting are selected based on the patient’s clinical manifestations.

**TABLE 1:** The Medical Effect of Herbal Ingredients used in Cyceon

Name	Drug nature according to *Corpus*	Source in *Corpus*	Note
Celery roots	Pass by stool better than its stalk	IV, *ΠΕΡΙ ΔΙΑΙΤΗΣ II*, LIV	Promote bowel movements
Dill	Hot and astringent, stop sneezing	IV, *ΠΕΡΙ ΔΙΑΙΤΗΣ II*, LIV	​
Rue	Pass better by urine than by stool, have a certain congealing quality, prevent poisoning	IV, *ΠΕΡΙ ΔΙΑΙΤΗΣ II*, LIV	Thickener
Mint	Warm, stop vomiting	IV, *ΠΕΡΙ ΔΙΑΙΤΗΣ II*, LIV	​
Coriander	Hot and astringent	IV, *ΠΕΡΙ ΔΙΑΙΤΗΣ II*, LIV	Help alleviate coughs
Fresh poppy	Binding, nourishing and strengthening	IV, *ΠΕΡΙ ΔΙΑΙΤΗΣ II*, XLV	With a strong cough-inhibition effect, fresh one is hotter than that stored for a long time
Basil	Dry, hot and astringent	IV, *ΠΕΡΙ ΔΙΑΙΤΗΣ II*, LIV	Relieve coughs, its dryness moderated after boiling
Lentil	Heating, its decoction has a laxative effect	IV, *ΠΕΡΙ ΔΙΑΙΤΗΣ II*, XLV, LIV	​

Through the analysis of the three cases presented above, it becomes evident that regimen was also used to cure diseases, and whether the ingredients are used as food or drug depends on the application scenarios and objects. Furthermore, Hippocrates’ medication practices remained centered around the theory of the four elemental qualities. Particularly in Case 3, the complexity of drug selection, compatibility, and dosage choices vividly demonstrates Hippocrates’ extensive clinical experience and emphasis on practical application. The elemental qualities of these substances and their effects on bodily excretion could only be refined through extensive clinical trials and continuous theoretical refinement. Moreover, the condition in Case 3 is equally intricate. A straightforward analysis based solely on the four elemental qualities would present a challenge: bile and phlegm simultaneously contain cold, hot, dry, and moist qualities, making further guidance through the four elemental qualities alone insufficient. Therefore, analyzing such complex conditions requires not only grounding in the natural philosophical logic of the four elemental qualities but also incorporating medical logic derived from clinical practice. This latter aspect is reflected in Hippocrates’ supplementation and refinement of the four humors theory through practical experience. Case 3 exemplifies his summary of the dynamic changes of the four humors within the body: The warm and moist blood is the earliest produced, being the freshest, most nutrient-rich, and most beneficial. As the body’s movements consume nutrients, the moist blood gradually transforms into dry and hot yellow bile. Over time, yellow bile cools and becomes dry and cold black bile, which is neither harmful nor beneficial. Subsequently, the non-nutrient black bile continues to age, absorbing moisture and depositing as cold and moist phlegm, ultimately being excreted from the body. This also manifests macroscopically as the transformation of elemental qualities across human life stages: children exhibit moist-warm properties, growing most rapidly; young adults display dry-warm traits as childhood moisture has dissipated; when growth ceases, individuals assume dry-cold characteristics; the elderly are prone to be moist, as fire has subsided while water continues to invade, causing dryness to vanish and the moisture brought by water to reside within (Hippocrates, 1931). Case 3 demonstrates that effective medication requires not only understanding the theory of the four elemental qualities and drug properties, but also comprehending the dynamic patterns of the four humors. This approach enables precise therapeutic interventions: replenishing blood deficient in moist-hot caused by excessive consumption, while simultaneously metabolizing and eliminating excess stagnant bile and phlegm. Thus, theoretical frameworks must be supplemented and refined according to practical needs, reflecting Hippocrates’ emphasis on practical application and his innovative exploration of integrating theory with practice.

Meanwhile, the extensive use of cyceon and wine as oral administration ways in the *Corpus* reflects Hippocrates’ cultural inheritance from the Homeric and Archaic period. The *Homeric Epics* contain the earliest records of cyceon as a medicinal formula in ancient Greece. In the *Odyssey*, the sorceress Circe, a master of concocting cyceon, served it made with barley meal, cheese, honey, Pramnian wine (οἴνῳ Πραμνείῳ)^11^, and a harmful drug to Odysseus’ companions, which made them forget their homeland (Homer, 1919). The consumption and preparation of wine also first appeared in the *Homeric Epics*, most commonly mixed with water, though there are also records of it being blended with honey, goat cheese, and barley meal (Homer, 1919; Homer, 1924). In *The Homeric Hymn to Demeter*, Metaneira initially offered honeyed wine to Demeter but was refused. Later, following the request of goddess, she served cyceon made with barley meal, water, and fresh pennyroyal (γλήχων, *Mentha pulegium* L.) (Foley, 1994) which may have been the basis for the ingredients and mythological origins of the ritual drink in the Eleusinian Mysteries.

In summary, regimen fully embodies the philosophical concepts of opposition, balance, harmony, and homeostasis. It not only demonstrates the pharmacological essence of the four elemental qualities in Hippocratic medicine at the practical level, but also reflects the medical idea of respecting facts and aligning theoretical explanations with practical applications. Furthermore, the pharmaceutical formulation employed showcase cultural inheritance from the Homeric and archaic period of ancient Greece, reflecting the practical continuity and innovation of classical medicine.

## 4 Hippocrates’ *pharmacon*: evacuative drugs and prescription

Evacuative drugs are narrowly defined medications with Hippocratic characteristics. As previously mentioned, some regimen drugs also exhibit mild laxative action. In contrast, evacuative drugs demonstrate more potent effects in the *Corpus*. The term “evacuation” primarily encompasses vomiting, purging, sweating, sneezing, and bleeding, aiming to eliminate harmful or excess substances. The most commonly used agents are purgatives and emetics. The *Aphorisms* states that defecation and vomiting are natural therapeutic functions. If the expelled substance is what should be expelled, the patient benefits; otherwise, it is harmful. Artificially induced vomiting and diarrhea are, in principle, the same as natural ones. Physicians should consider factors such as season, region, patient age, and condition to ensure the treatment is appropriate (Hippocrates, 1931).

Unlike regimen drugs, evacuants exhibit limited connections with philosophical concepts such as the Four Elemental Qualities and the Four Humors due to their restricted functions. The varieties and amount of them are far more restricted than that of regimen drugs, manifesting as a material dependence on specific herbal species. Nevertheless, Hippocrates still explored prescriptions to minimize such dependence, aiming to address complex and variable clinical scenarios and further expand the buffer zone between therapeutic gradients. This demonstrates that evacuative drugs are more closely linked to clinical practice, reflecting the exploration of medicinal plants and continuous innovation in formulary development. It also highlights Hippocrates’ emphasis on practical application and his pursuit of diversified drug use.

### 4.1 Hellebore

Hellebore (ἐλλέβορος) is the most commonly used evacuative herb in the *Corpus*, encompassing at least two species today: black hellebore (μέλας ἐλλέβορος, *Helleborus* sp.)^12^ and white (false) hellebore (λευκός ἐλλέβορος, *Veratrum album* L.).

Black hellebore was commonly used for purging. According to *De materia medica*, black hellebore primarily grows in Anticyra, Mount Helicon, Mount Parnassus, and Aitolia, with the highest quality found in Anticyra and Mount Helicon (Dioscorides, 2005). Hippocrates also mentioned his optimal formula for black hellebore, which was gentler than single herb and more harmoniously combined: using black hellebore as the main ingredient, mixed with daucus (δαῦκος, perhaps *Athamanta cretensis* L.), hartwort (σέσελι, *Tordylium officinale* L.), cumin (κύμινον, *Cuminum cyminum* L.), and anise (ἄνησον, *Pimpinella anisum* L.) (Hippocrates, 1923b).

White hellebore, not belonging to *Helleborus* but *Veratrum*, namely, false hellebore, is mainly used for emesis and also has a sneezing effect when applied to the nasal cavity. Its emetic effect is extremely potent, and Dioscorides mentioned that the suppository made from it and vinegar could even induce vomiting. White hellebore from Anticyra is the best, in addition to those from Galatia, France, and Cappadocia (Dioscorides, 2005). In TCM, Lilu (藜芦, *Veratrum nigrum* L.) has a similar emetic effect, which is of the same genus with white hellebore. It was first recorded in *Shennong Bencaojing*, classified as the third grade, used to vomit out Wind-Phlegm and treat disease caused by worm toxin (Hongjing, 1994; Shusheng, 2009; Xingyan, 2017). Li Shizhen commented, “It is incompatible to Congbai. Incessant emesis will be caused by taking it, and a decoction of Cong will stop it” (Shizhen, 2011; 2021).

### 4.2 *Euphorbia* sp.

Wild purslane (purple spurge, πεπλιον, *Euphorbia peplis* L.) has effects similar to black hellebore and is another single-herb purgative commonly used by Hippocrates. Its best combination is to be taken with silphium (σίλφιον). Hippocrates believed that black hellebore had a stronger purgative effect than wild purslane and were more favourable to the crisis, while wild purslane was superior to black hellebore in treating flatulence, and both could relieve pain. In China, the spurge (大戟, *Euphorbia pekinensis* Rupr.) is commonly used in the ancient times, first recorded by *Shennong Bencaojing*, classified as a third-grade herb in it, “[mainly used] to treat [disease caused by] worm toxin, various edema, abdominal fullness and sharp pain, accumulation, aggregation, wind stroke, cutaneous pain and vomiting” (Hongjing, 1994; Xingyan, 2017). The spurge bitter and cold, could promote diuresis and relieve constipation, with a strong purgative effect similar to Kansui (甘遂, *Euphorbia kansui* Liou ex S. B. Ho) but slightly weaker (Shusheng, 2009).

Sun spurge or *Zeqi* (τιθύμαλλος ἡλιοσκόπιος, 泽漆, *Euphorbia helioscopia* L.), an another herb of *Euphorbia* sp. both used in ancient Greece and China, widely distributed in Europe, North Africa, and East Asia. In the *Corpus*, sun spurge was included in the terms *τιθύμαλλος* or *μηκώνιον*, which are commonly used to refer to various *Euphorbia* sp. (Hippocrates, 1988). According to Dioscorides’ records, its foliage turns as the sun sets, hence it was named “solar observer (ἡλιοσκόπιος)”^13^. Sun spurge is rich in milky juice. A quantity of 2 obols (ὀβολός)^14^ of the milky juice mixed with watered sour wine could purge the lower abdomen, expelling phlegm and bile; while mixed with hydromel, it is emetic. Topical application of the milky juice can also remove warts that spread under skin. The root of sun spurge purges too when 1 drachma (δραχμή)^15^ of it is sprinkled with hydromel and drunk (Dioscorides, 2005). Sun spurge in China was also first recorded in the *Shennong Bencaojing*: “*Zeqi*, bitter in taste, slightly cold in property, is mainly used for skin heat, retention of fluid in the abdomen, dropsy of the limbs, face and eyes, as well as impotence in men” (Hongjing, 1994; Xingyan, 2017). Li Shizhen commented “*Zeqi* purges fluid-retention and has a similar functions to spurge.” (Shizhen, 2011; 2021) Whether for purging or treating skin warts, the clinical effects of sun spurge are mutually corroborated by the medical texts from both ends of the ancient world.

### 4.3 Other compound medicine

In addition to single-herb formulations such as black hellebore, white hellebore, and *Euphorbia* sp., the *Corpus* also documents compound remedies composed of substances commonly found in ancient Greek daily life, which were used for emesis or purging. These remedies may have slightly weaker efficacy compared to the aforementioned potent drugs, but exhibited stronger evacuative effects than regimen drugs. Serving as a transitional gradient between regimen and *pharmacon*, they further enriched the application scenarios of evacuative drugs. For instance, *Regimen in Health* (*ΠΕΡΙ ΔΙΑΙΤΗΣ ΥΓΙΕΙΝΗΣ*) mentions an oral emetic prescription”: half a cotyle of hyssop (ὕσσωπος, *Micromeria graeca* (L.) Benth. ex Rchb.) compounded with a chous (χοῦς)^16^ of water, pouring in vinegar and adding salt, and let the patient drink this.” It also describes two purgative clysters: one for people inclined to fatness and moistness, prepared with brine or sea-water; the other for those inclined to dryness, leanness and weakness, formulated with milk (γάλα) or water boiled with chick-peas (ἐρέβινθος, *Cicer arietinum* L.) or other nutrient-rich liquids (Hippocrates, 1931).

At this point, we can summarize the panorama of Hippocrates’ therapeutic pharmacal gradient (Table 2) from medical practice to enrich his “regimen–*pharmacon*” classification: based on clinical requirements, Hippocrates categorized regimen clinically into two types according to the objects of regimen—for the health and for patients. According to the intensity of evacuative effects, he also established a gradient of evacuation (*pharmacon*) therapies, including mild evacuation, moderate evacuation, and strong evacuation. Among these, mild evacuation occupies a position between regimen and evacuation therapy in terms of medical treatment, but it is classified as a regimen drug in pharmacal categorization, as it is an additional factor to consider when formulating regimen prescriptions.

**TABLE 2:** Hippocrates’ therapeutic and pharmacal gradient.

Classification in *corpus*	Medical treatment	Drug categorization
Regimen	Dietary regimen	Food (not as drug)
	Pharmacal regimen	Drugs for regimen
	Mild evacuation (laxative)	
*Pharmacon*	(Moderate) evacuation (purgative)	Drugs for evacuation
	Strong evacuation (purgative)	

## 5 Conclusion

Hippocrates’ pharmacology is not a simple linear narrative from theory to practice, but rather a continuous and iterative medical system derived from the cognition of drug properties and clinical experimental verification. This article systematically organizes and analyzes the medical theories and pharmacal practices related to pharmacology in the *Corpus*, extracting the system of Hippocratic pharmacology and revealing the connections among philosophical theories, pharmacal gradient and clinical medication.

The uppermost layer of the system is philosophical theories serving as a theoretical guidance for clinical medication. The theory of four humors is undoubtedly Hippocrates’ innovation and composing the core of his physiology. The four elemental qualities, as the underlying logic of Hippocrates’ physiology, pathology, and pharmacology, reflects the continuity and development of Hippocratic medical philosophy from ancient Greek natural philosophy. The middle layer of the system serves as a transitional bridge linking metaphysical concepts with practical applications, which initially stemmed from the “regimen–*pharmacon*” classification in *Corpus*, functioning as an engineering framework that guides clinical medication based on upper-layer theories. Last but not least, the clinical practice constitutes the true medical core of Hippocrates, as the ultimate goal of all the theories is to heal and save lives. Through clinical feedbacks, Hippocrates continuously improved the middle-layer framework and upper-layer theories, such as refining treatment gradients, transforming of four humors, changes of age-related physiological properties, and supplementing and correcting seasonal regimen theory.

In summary, Hippocrates demonstrated inheritance and innovation in both philosophical theory and pharmacal practice. Through a theory-framework-practice system, this paper provides a detailed analysis of the relationship between medical philosophy and clinical medication of the Hippocratic school. A hierarchical therapeutic framework is constructed based on *Corpus*, encompassing dietary regimen, pharmacal regimen, and mild, moderate, potent evacuation, as well as their corresponding foods or drugs. Notably, Hippocrates’ advocacy for regimen and his criticism for his contemporaries’ overreliance on evacuants reflect a medical philosophy akin to “treating disease before it occurs” and the virtue of avoiding excessive treatment. His exploration of medicinal plants and drug formulations also demonstrates his emphasis on practicality and innovation in pharmacal application. However, this study still remains the following limitations. One is the challenge for readers to clarify the system and the gradient framework in it, which arises from the trial structure of the article and complex connections between philosophy and clinic; the other is the reproductive and surgical medicine are not covered, as they have characteristics respectively, only major internal medicine being discussed in this study.

## Data Availability

The original contributions presented in the study are included in the article/supplementary material, further inquiries can be directed to the corresponding author.

## Footnotes

1 The “crisis” is a pivotal concept in Hippocratic medicine, referring to one or several days during the disease course that are exceptionally critical. Intervention and treatment in these critical days play an exceptionally important role in disease cure.

2 Polybius, the son-in-law of Hippocrates, succeeded him as the leader of the Kos School after Hippocrates left Kos for Thessaly. Note that this Polybius should not be confused with the Hellenistic historian Polybius.

3 The term “elemental component” denotes “primary substance,” where “elemental” signifies natural indivisible “one”. In Hippocrates’ original text, it was used to denote water, fire, earth, air, cold, heat, dryness, moisture, blood, phlegm or bile. The cold, heat, dryness, and moisture were classified as “four elemental qualities,” forming philosophical group terminologies alongside “four elements” and “four humors.” However, “four elemental qualities” also implies four “properties” in addition to “substances,” reflecting the dual nature of “substance and property” in ancient Greek philosophy. Particularly in Hippocrates’ humor theory, the term leans more toward “property.”

4 Some scholars, citing Aristotle’s analysis of the “veins” in Chapter XI of *Nature of Man*, argue that this work was authored by Polybius, a student and son-in-law of Hippocrates. However, Jones contends that the current version consists of both Hippocrates’ original text and Polybius’ later additions (Hippocrates, 1931).

5 Regimen refers to the practice of regulating the body’s cold, heat, dryness, and moisture through appropriate intake (food and drug) and exercise, thereby maintaining or restoring health (Hippocrates, 1931). This study focuses on pharmacy and therefore excludes the regulation of exercise.

6 Patient compliance refers to the patient’s adherence degree to medical treatment and instructions, which can also be understood as the degree of cooperation between the patient and the physician. Drug compliance specifically manifests as the patient’s adherence degree to the prescribed dosage, duration, and administration way of the medication. Lack of compliance may lead to treatment failure, disease recurrence, adverse reactions, or even life-threatening consequences. Examples of low compliance include children’s natural aversion to bitter medications.

7 Cyceon, the most simple one is made with barley meal only added to water, which is cooling in nature yet nutritious.

8 The *Corpus* contain several recipes for adding wine and honey to cyceon, which should all be considered as cyceon.

9 The cotyle, an ancient Greek unit of liquid measures, is approximately equivalent to 273.6 mL today.

10 The oxybaphon, an ancient Greek unit of liquid measures. Four oxybapha is equivalent to 1 cotyle, with 1 oxybaphon approximately equaling 68.4 mL today.

11 Pramneios was a premium, relatively strong red wine in ancient Greek mythology.

12 Jones annotated “μέλας ἐλλέβορος” as “*Helleborus niger*”, Poter as “*Helleborus cyclophyllus*”. In modern English, “black hellebore” refers to “*Helleborus niger* L.”. Some (particularly early) scholars considered the “μέλας ἐλλέβορος” in the *Corpus* to be this species; however, more scholars now believe that “μέλας ἐλλέβορος” is “*Helleborus cyclophyllus* (A. Braun) Boissier”. Additionally, some scholars argue that “μέλας ἐλλέβορος” encompasses several species of the genus *Helleborus*. See (Hippocrates, 1923b; 1988; Penn, 1994) https://en.wikipedia.org/wiki/Helleborus_cyclophyllus, last edited on 1 December 2024, at 17:29 (UTC).

13 The term is composed of “ἥλιος (sun)” and “σκοπέω (to watch),” “Ἥλιος” also being the name of the ancient Greek sun god Helios.

14 1 obol is approximately 0.568 g (6 obols equal 1 drachma). Since the unit was based on coin weight, ancient Rome had a lighter weight standard compared to ancient Greece.

15 1 drachma is approximately 3.411 g.

16 The chous, an ancient Greek unit of liquid measures, was equivalent to 12 cotylai, approximately 3.283 L today.
